# The Effect of Vitamin D Supplementation on Glycemic Control in Type 2 Diabetes Patients: A Systematic Review and Meta-Analysis

**DOI:** 10.3390/nu10030375

**Published:** 2018-03-19

**Authors:** Xinyi Li, Yan Liu, Yingdong Zheng, Peiyu Wang, Yumei Zhang

**Affiliations:** School of Public Health, Peking University Health Science Center, 38 Xueyuan Road, Beijing 100191, China; 1310306218@pku.edu.cn (X.L.); shyneeliu@bjmu.edu.cn (Y.L.); ydzheng@bjmu.edu.cn (Y.Z.); wpeiyu@bjmu.edu.cn (P.W.)

**Keywords:** vitamin D, type 2 diabetes, glycemic control, meta-analysis

## Abstract

Observational studies have indicated an inverse association between vitamin D levels and the risk of diabetes, yet evidence from population interventions remains inconsistent. PubMed, EMBASE, Cochrane Library and ClinicalTrials.gov were searched up to September 2017. Data from studies regarding serum 25(OH)D, fasting blood glucose (FBG), hemoglobin A1c (HbA1c), fasting insulin and homeostasis model assessment of insulin resistance (HOMA-IR) were pooled. Twenty studies (*n* = 2703) were included in the meta-analysis. Vitamin D supplementation resulted in a significant improvement in serum 25(OH)D levels (weighted mean difference (WMD) = 33.98; 95%CI: 24.60–43.37) and HOMA-IR (standardized mean difference (SMD) = −0.57; 95%CI: −1.09~−0.04), but not in other outcomes. However, preferred changes were observed in subgroups as follows: short-term (WMD_FBG_ = −8.44; 95%CI: −12.72~−4.15), high dose (WMD_FBG_ = −8.70; 95%CI: −12.96~−4.44), non-obese (SMD_Fasting insulin_ = −1.80; 95%CI: −2.66~−0.95), Middle Easterners (WMD_FBG_ = −10.43; 95%CI: −14.80~−6.06), baseline vitamin D deficient individuals (WMD_FBG_ = −5.77; 95%CI: −10.48~−1.05) and well-controlled HbA1c individuals (WMD_FBG_ = −4.09; 95%CI: −15.44~7.27). Vitamin D supplementation was shown to increase serum 25(OH)D and reduce insulin resistance effectively. This effect was especially prominent when vitamin D was given in large doses and for a short period of time, and to patients who were non-obese, Middle Eastern, vitamin D deficient, or with optimal glycemic control at baseline.

## 1. Introduction

Type 2 diabetes (T2D) has become a global health care problem. In 2016, there were 38 million diabetes patients worldwide, with 2.8 million years lived with disability [1]. Efforts have been devoted to finding innovative approaches for diabetes prevention and treatment, and a recent focus has been on vitamin D supplementation. Observational studies have indicated an association between vitamin D deficiency and the onset and progression of T2D as well as future macrovascular events [2,3,4,5,6]. Moreover, in vivo and in vitro studies have proposed potential roles of vitamin D in glucose metabolism, e.g., stimulating insulin secretion via the vitamin D receptor on pancreatic β cells; modulating immune responses and lowering systematic inflammation; and reducing peripheral insulin resistance through vitamin D receptors in the muscles and liver [7,8,9]. However, evidence from interventional studies at a population level have been inconclusive. Recently, three meta-analyses observed no benefits of vitamin D supplementation on glycemic indices and insulin resistance except for a modest reduction of hemoglobin A1c (HbA1c) (0.32–0.39%), although they did not separate intramuscular injections from oral supplementation, or fortified food from nutrient supplementation alone [10,11,12]. Additionally, co-supplementation of calcium plus vitamin D, versus a control group given vitamin C further introduced more heterogeneity in the studies. On the contrary, Mirhosseini et al. found significant positive effects of vitamin D supplementation on fasting blood glucose (FBG), HbA1c and a homeostasis model assessment of insulin resistance (HOMA-IR), yet it calculated one study with considerable influence twice in the pooled analysis and combined results reported as median and interquartile range together with those reported as mean and standard deviation [13].

The aim of the current review was to evaluate the effects of oral vitamin D supplementation on glycemic control in T2D patients compared with a placebo, and to assess various factors’ influences on supplementation effects.

## 2. Methods

This systematic review was performed according to the Preferred Reporting Items for Systematic Reviews and Meta-analysis (PRISMA) statement [14]. 

### 2.1. Selection of Studies

Studies were regarded as eligible if they (1) were randomized controlled trials that evaluated the glycemic effect of vitamin D supplementation in T2D patients; (2) used oral vitamin D formulations containing cholecalciferol or ergocalciferol; (3) reported at least one of the following primary outcomes of interest: serum 25(OH)D concentration, FBG, HbA1c, HOMA-IR and fasting insulin; and (4) had a trial length ≥ 8 weeks.

Exclusion criteria were as follows: (1) intramuscular delivery of vitamin D with different absorptions between oral and intramuscular routes; (2) studies involving participants with type 1 diabetes, gestational diabetes or conditions that could potentially alter vitamin D metabolism (e.g., chronic kidney disease Stage 4 or higher, or hyperparathyroidism); (3) studies involving participants < 18 years; (4) studies in which non placebo controls, such as calcium, were allowed when given to both groups; and (5) observational studies, review articles, case reports, editorials and poster abstracts. 

### 2.2. Data Sources and Search Strategy

We searched databases including PubMed, EMBASE and Cochrane Central Register of Controlled Trials (CENTRAL) from inception to September 2017 to identify relevant studies published in English. We also searched NIH’s clinical trials registry (www.clinicaltrials.gov) for unpublished but completed trials. The reference lists of the index reviews were also reviewed to identify additional eligible studies. The following search strategy was run in PubMed and tailored to each database when necessary: (randomized controlled trial OR controlled clinical trial OR random OR clinical trial OR controlled trial OR RCT NOT review NOT animal) AND (vitamin D OR vitamin D_2_ OR vitamin D_3_ OR cholecalciferol OR ergocalciferol OR alphacalcidol OR alfacalcidol OR paricalcitol OR doxercalciferol OR calcitriol OR 25-Hydroxyvitamin D) AND (diabetes OR diabetes mellitus OR T2DM OR hyperglycemia OR hyperglycaemia OR glucose OR HbA1c OR glycated hemoglobin OR insulin resistance OR insulin sensitivity OR HOMA OR glucose homeostasis OR insulin secretion OR insulin OR beta-cell function OR glycemic control OR glycemic control OR glucose tolerance OR glucose metabolism).

### 2.3. Data Extraction and Risk of Bias Assessment

Pairs of independent reviewers screened the titles and the abstracts of each study prior to full text screening of candidate studies. Any discrepancies in terms of the decision on a given study were dealt with via discussion and, if necessary, arbitration by a third reviewer. For all included studies, two reviewers independently extracted information regarding basic information (authors, year, country, sample size, attrition rate), participants’ characteristics (gender, age, body mass index (BMI), ethnicity), intervention features (treatment, type, dose, and therapy duration) and results (baseline and post-test serum vitamin D, FBG, HbA1c and fasting insulin). For studies assessing the differences between a placebo, a vitamin D, a calcium and a vitamin D plus calcium group, we only used the vitamin D and placebo groups. 

Two reviewers independently assessed the risk of bias of trials according to the Cochrane risk of bias assessment tool and assigned “high” or “low” or “unclear” to the following items: random sequence generation; allocation concealment; blinding of participants and personnel; blinding of outcome assessment; selective reporting; and other sources of bias [15].

### 2.4. Data Analysis and Rating Quality of Evidence

We combined the pre- and post-changes of each outcome with the random effects model due to diverse participant demographics and intervention characteristics; this was reported with weighted mean differences (WMD) for scales that were the same, or standardized mean differences (SMD) otherwise. Substantial heterogeneity was indicated as *p* < 0.1 in the χ^2^ test and an I^2^ > 50% [16].

Sensitivity analyses were completed to detect the robustness of the statistical results and analyze possible sources of heterogeneity, using an alternative summary statistic (standardized versus weighted mean difference) and statistical model (fixed versus random effects model), excluding studies on a one by one basis, followed by the exclusion of those with a high risk of bias. 

When an adequate number of studies ensuring the power of tests, Funnel plots and Egger’s test had been conducted, to identify any publication bias. Subgroup analyses were performed to explore impacts of certain characteristics: supplementation dose, duration, ethnicity, BMI status (normal ≤ 24.9 kg/m^2^, overweight 25–29.9 kg/m^2^, obese ≥ 30 kg/m^2^) [17], baseline vitamin D status (deficiency < 50 nmol/L, insufficiency 50–75 nmol/L, sufficiency > 75 nmol/L) [18] and baseline HbA1c condition. Univariate and multivariate meta-regressions, if possible, were performed to detect whether %female, BMI, serum vitamin D concentration, FBG, or HbA1c at baseline were associated with the results. All data were analyzed using Stata 14.0 and Cochrane Review Manager (RevMan) 5.3.

The GRADE approach was used to rate the quality of evidence for each outcome. The strength of the evidence was categorized as high, moderate, low or very low [19].

## 3. Results

**Figure 1:**
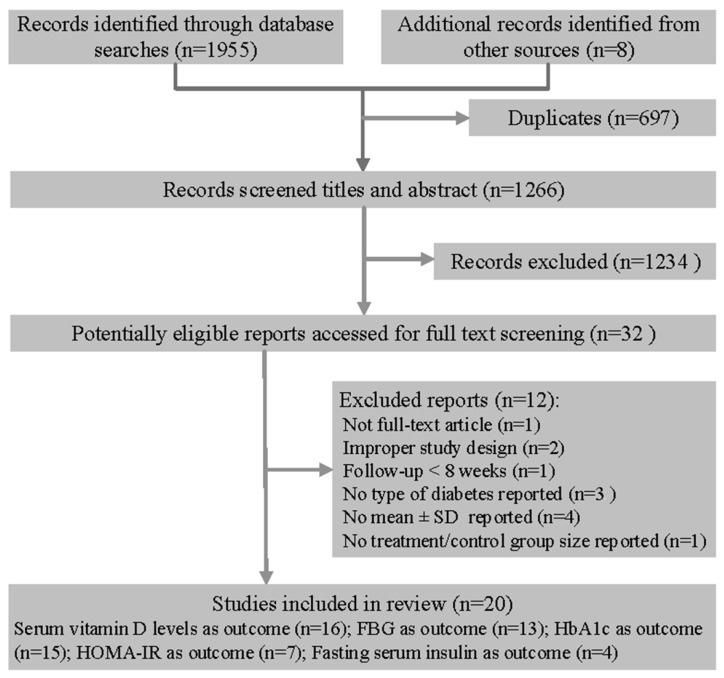
Flow diagram of search and selection of studies.

Of the 1963 articles, 32 remained as potentially eligible after title and abstract screening; of these, 20 randomized controlled trials involving 2703 participants were included in the review (Figure 1) [20,21,22,23,24,25,26,27,28,29,30,31,32,33,34,35,36,37,38,39]. 

### 3.1. Study Characteristics

Characteristics of all 20 studies are presented in Table 1. Among those, eight enrolled Middle Easterners [21,22,23,29,31,34,36,38], four enrolled individuals from other Asian countries [26,32,33,39], seven enrolled other ethnicities [20,24,25,27,28,35,37] and the one was conducted in 33 countries [30], with participants mainly from 48 to 67 years old. The intervention duration ranged from 2 to 6 months (median was 3 months). Vitamin D_3_ was used in 17 studies and vitamin D_2_ in one study [35], while the other two failed to report the type in detail [31,38]. Only two trials applied vitamin D and calcium co-supplementation (same doses of calcium also given to control groups) [32,33]. The other studies provided vitamin D supplementation alone.

### 3.2. Risk of Bias

**Figure 2:**
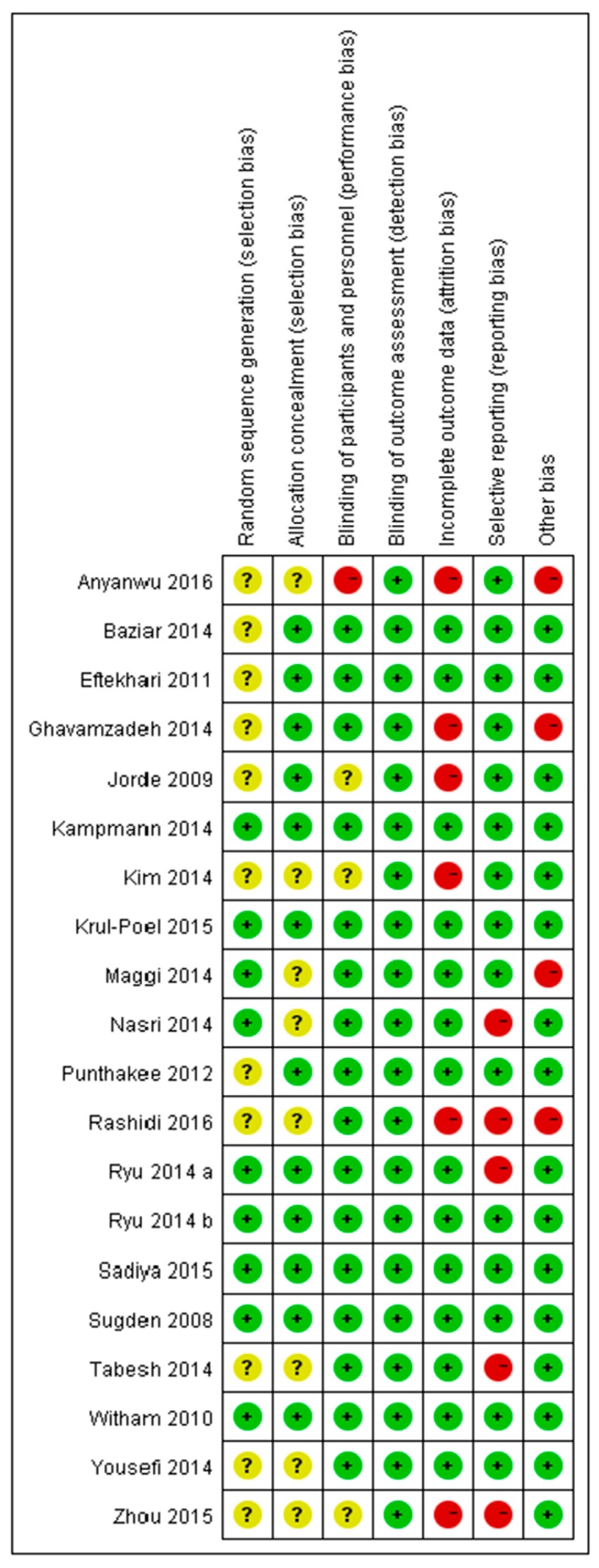
Risk of bias assessment of the included studies using the Cochrane Collaboration tool across seven domains. Risk of bias levels: low (green), unclear (yellow), high (red).

The risk of bias assessments of the included studies are summarized in Figure 2. Ten, six and four studies were categorized as having high, low and unclear risks of bias, respectively. Attrition and reporting bias were primary sources of bias.

### 3.3. The Effect on Serum Vitamin D Level

**Figure 3:**
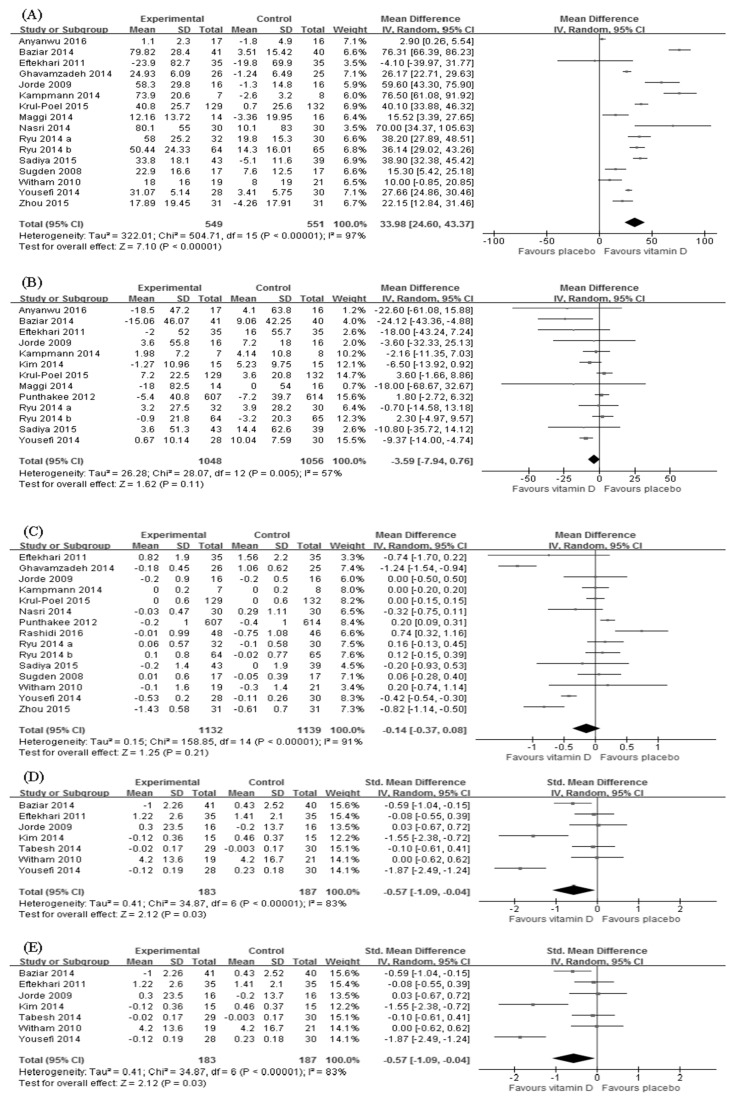
Effect of vitamin D supplementation on (**A**) serum vitamin D level; (**B**) fasting blood glucose; (**C**) hemoglobin A1c; (**D**) homeostasis model assessment of insulin resistance and (**E**) fasting insulin. (The size of box represents the weight of each study, and the lateral tips of diamond shows the confidence interval of the pooled result.)

Sixteen trials (*n* = 1284) reported the change in serum vitamin D level after the intervention [20,21,22,23,24,25,27,28,29,32,33,34,35,37,38,39]. There was a significant increase in the serum vitamin D level in the vitamin D supplementation group (WMD = 33.98; 95%CI: 24.60–43.37; *p* < 0.001) (Figure 3A), albeit with substantial heterogeneity (*p* < 0.001; I^2^ = 97%). Subgroup analyses suggested similar findings in all subgroups except for participants with sufficient vitamin D prior to the intervention (Table 2).

### 3.4. The Effect on FBG

Thirteen trials (*n* = 2198) measured the effect of vitamin D supplementation on FBG [20,21,22,24,25,26,27,28,30,32,33,34,38]. Overall, we observed no difference in FBG reduction between intervention and control groups (WMD = −3.59; 95%CI: −7.94–0.76; *p* = 0.11). The heterogeneity was moderate (*p* = 0.005; I^2^ = 57%) (Figure 3B). However, vitamin D supplementation produced significant decreases in the following subgroups: Middle Easterners, dose > 2000 IU/day, study duration ≤ 3 months and baseline vitamin D deficient (Table 2).

### 3.5. The Effect on HbA1c

Changes in HbA1c was assessed in 15 trials (*n* = 2454) [22,23,24,25,27,29,30,31,32,33,34,35,37,38,39]. Compared with controls, the difference in HbA1c reduction was insignificant (WMD = −0.11; 95%CI: −0.35–0.13; *p* = 0.38) (Figure 3C) and had high heterogeneity (*p* < 0.001; I^2^ = 92%). Nevertheless, effects shown in other ethnicities and obesity subgroups were significantly in favor of the control group (Table 2). 

### 3.6. The Effect on HOMA-IR

The effect of vitamin D supplementation on HOMA-IR was evaluated in seven trials (*n* = 409) [21,22,24,26,36,37,38]. We found a larger reduction of HOMA-IR in intervention groups than in placebo groups (SMD = −0.57; 95%CI: −1.09~−0.04; *p* = 0.03) although the heterogeneity was significant (*p* < 0.001; I^2^ = 83%) (Figure 3D). Similar results were demonstrated in subgroups of duration ≤ 3 months, other Asians, normal BMI, and baseline vitamin D deficient (Table 2).

### 3.7. The Effect of Fasting Insulin

Only four (*n* = 364) out of 20 randomized controlled trials measured fasting insulin [21,25,26,38]. Results showed no significant effect of vitamin D supplementation on fasting insulin (SMD = −0.84; 95%CI: −2.27–0.60; *p* = 0.25) (Figure 3E). The heterogeneity was relatively high (*p* < 0.001; I^2^ = 93%). However, preferred changes were manifested in the following subgroups: other Asians, normal BMI, vitamin D dose ≤ 2000 IU/day and baseline HbA1c > 7%. In contrast, other ethnicities and obesity subgroups showed preferred changes that were significantly in favor of the control group (Table 2). 

### 3.8. Meta-Regression

The univariate meta-regression suggested that a higher baseline FBG was associated with a better effect of vitamin D supplementation in FBG (*p* = 0.04). However, this association vanished after adjusting for % female, BMI, baseline serum vitamin D and HbA1c in the multivariate meta-regression (*p* = 0.10).

### 3.9. Sensitivity Analyses

Findings regarding serum vitamin D levels and glycemic indices basically remained robust in the sensitivity analysis. However, the significant effect of vitamin D supplementation on HOMA-IR disappeared when excluding Baziar et al. [21], Yousefi et al. [38], Kim et al. [26] and trials carrying a high risk of bias [24,26,36]. On the contrary, the improvement of FBG became significant after Kampmann et al. and Krul-Poel et al. were excluded and an alternative summary statistic was used [25,27]. In addition, excluding Yousefi et al. from the meta-analysis of FBG resulted in a decrease in heterogeneity from 57% to 33% [38].

### 3.10. Publication Bias

**Figure 4:**
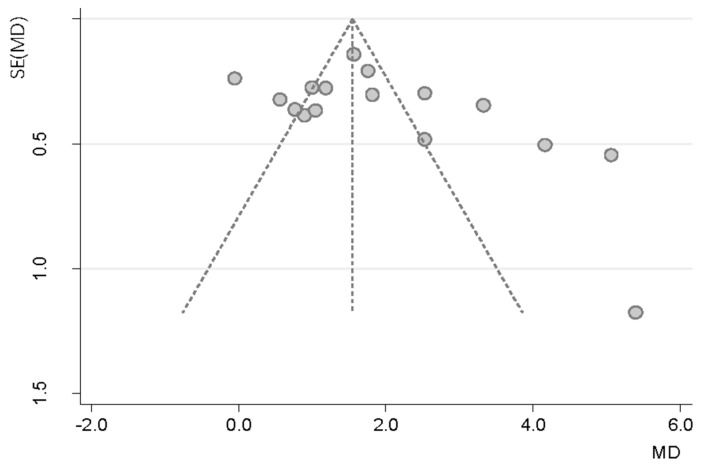
Funnel plot of serum vitamin D changes in patients with type 2 diabetes receiving vitamin D supplementation based treatment versus controls in 16 randomized controlled trials (Each circle represents a study, with the y-axis representing the standard error of the study and the x-axis meaning the weighted mean difference of it. The funnel-shaped distribution is created by studies with high precision plotted near the average, and studies with low precision spread evenly on both sides of the average. Deviation from this shape indicates publication bias).

The funnel plot and statistical test showed no evidence of a publication bias (Figure 4; Egger’s test: *p* = 0.81; 95%CI: −5.39–6.77).

### 3.11. Quality of Evidence

Table 3 presents the quality of evidence by outcome, assessed with the GRADE system. Evidence quality was classified as low for serum vitamin D level and HOMA-IR, and very low for FBG, HbA1c and fasting insulin.

## 4. Discussion

This meta-analysis found that compared with placebo, oral vitamin D supplementation yielded better effects on HOMA-IR in T2D patients, although the result was not very robust when excluding studies one by one, and it was subject to substantial heterogeneity partially from the diversity between durations, ethnicities, BMIs and baseline vitamin D statuses across the studies. In contrast, we observed no benefit of vitamin D supplementation in regard to improving FBG, HbA1c and fasting insulin. However, despite the overall null finding, characteristics of the intervention and population greatly influenced the effects, and significant positive effects emerged in several subgroups.

In terms of the intervention, daily doses of more than 2000 IU/day were consistently associated with a higher post-test vitamin D status and larger improvement of glycemic indices. The appropriate dose of vitamin D to achieve non-skeletal benefits still remains unclear. Some observational studies indicated that supraphysiological dosing of vitamin D could be harmful; however, the most common dosing of supplementation, 2000 IU/day, was much lower than that, as well as than the debated dosing required for efficacy, 4000 to 5000 IU/day [13,40]. Under these conditions, the benefits of higher doses seemed reasonable since high doses increased the chances of correcting vitamin D deficiency or achieving favorable levels of serum 25(OH)D, confirmed by relatively higher 25(OH)D levels in the high dose subgroup after intervention (78.52 versus 75.01 nmol/L in the low dose subgroup). 

The impact of intervention duration was more ambiguous. Contrary to the findings of Lee et al. and Mirhosseini et al. that longer durations of supplementation were associated with larger reductions in glycemic control, which was possibly due to the 2–3 month lifespan of HbA1c [11,13], our results were in line with Krul-Poel et al. and suggested the effects of vitamin D supplementation were mainly manifested in durations shorter than 3 months [10]. A potential explanation was the difference in doses. Actually, the average dose of studies included in the short-term subgroup was much higher than that in the long-term subgroups (3000 versus 1833 IU/day). In addition, the worsening of diabetic conditions over time could also attenuate the effects of a long-term intervention. 

Apart from intervention features, several characteristics of participants also played important roles in modifying the efficacy of supplementation. First, significant reductions in FBG and HOMA-IR were observed in patients with vitamin D deficiency but not in patients with vitamin D insufficiency or sufficiency. Similarly, we found that even though 25(OH)D increased significantly in all subgroups, the highest improvement came in vitamin D deficient patients. This might be responsible for advantages in FBG and HOMA-IR shown in the vitamin D deficient group and could be because in the sufficient group the excess vitamin D from supplementation was stored in adipose tissue rather than triggering a further increase in serum level.

Second, vitamin D supplementation produced different effects among various ethnicities with a similar trend: Middle Easterners showed the biggest reduction, the other Asians the second, and other ethnicities had the smallest preferred changes. Incongruent results in subgroup analyses of HOMA-IR and fasting insulin were due to the limited number of studies that measured these variables. As Wang et al. demonstrated that vitamin D-binding protein polymorphism was associated with increased susceptibility to T2D in Asians, but not in Caucasians; this might explain the heterogeneous responses between Asians and the other groups [41]. In addition, studies found that populations with darker skin color and cultural preferences toward less exposure to the sun, which matched the profile of Middle Easterners, were at a higher risk of vitamin D deficiency, and that, in turn, was associated with better effects from supplementation [42,43]. However, none of the studies we included in the analysis were based on American participants’ data. Thus, the supplementation effect of vitamin D in this population warrants further investigation in the future.

Third, better glycemic parameters appeared in non-obese patients. This observation is somewhat opposite to previous studies that suggested that BMI was negatively associated with serum 25(OH)D concentration and that supplementation would be especially helpful in obese patients [44]; however, a recent meta-analysis supports our finding that the obese population, even with an inadequate vitamin D status, does not benefit more from supplementation beacause of doses trapped in their fat mass [12,13]. However, our data suggest a different mechanism because although the entry level baseline vitamin D status of the obese subgroup was slightly lower than the non-obese subjects’ baseline status (40.14 vs. 45.05 nmol/L), the relationship was reversed in the post-test analysis (obese 89.96 versus non-obese 75.96 nmol/L), in contrast to the previously published hypothesis.

Finally, optimal baseline glycemic control, HbA1c ≤ 7% to be specific, was associated with preferred effects of vitamin D supplementation on serum 25(OH)D levels, FBG and HbA1c. The HOMA-IR and fasting insulin data did not support this finding, but the fact that they were based on a small number of studies reduced their credibility. Nevertheless, Krul-Poel et al. and Soric et al. reported that significant improvements in glucose metabolism were only manifested in patients with poor glycemic control at baseline [10,45]. While they used different cutoffs (HbA1c ≥ 8% and 9% respectively), applying alternative cutoffs did not change the results in this meta-analysis. Further investigations are required to clarify this association and explore the underlying explanation for it.

To our knowledge, this meta-analysis is unique because it evaluates the effects of vitamin D supplementation on beta cell function as measured by fasting insulin. In addition, our analysis takes into account the most recent years of publications on vitamin D and type 2 diabetes, thereby expanding our appreciation of fasting blood glucose, hemoglobin A1c and HOMAR-IR by encompassing the evidence base related to type 2 diabetes. We also used the GRADE system to assess the quality of the evidence. In addition, we conducted a comprehensive exploration of the influential factors on the supplementation effects and identified some important ones, like ethnicity, BMI, baseline vitamin D status and HbA1c. This might encourage further studies to confirm the influence and mechanisms of these factors and allow the provision of supplementation with appropriate timing and in appropriate subpopulations. Moreover, the other strength of our study is that we only included randomized controlled trials with oral vitamin D supplementation, mainly cholecalciferol; thus, the relative uniformity of the study design and formulation of vitamin D reduced the overall heterogeneity. However, heterogeneity was still significant owing to the various lengths, doses, and participants involved in the studies. Other limitations include the fact that most studies did not assess effects of sun exposure, dietary intake and physical exercise, which may also have influenced vitamin D status. In addition, the use of antidiabetic medication or insulin therapy might also mask the benefits of vitamin D, especially in studies that allowed medication adjustment during the intervention period. Last, some trials included had relatively small sample sizes and short intervention durations.

## 5. Conclusions

Oral vitamin D supplementation has shown better effects in enhancing serum 25(OH)D levels and reducing insulin resistance compared with placebos among type 2 diabetes patients. However, it did not appear to influence FBG, HbA1c and fasting insulin levels. Large dosage, short-term vitamin D supplementation was most likely to yield preferred changes in vitamin D deficient, non-obese groups, Asians, especially Middle Easterners, and patients with optimal glycemic control at baseline. Additional large well-designed studies with longer duration are required to further clarify the impacts of BMI and baseline HbA1c.

## Figures and Tables

**Table 1:** Characteristics of studies included in the meta-analysis.

Author Year	Country	Participants (T/C ^1^)	Female (%)	Attrition Rate (%)	Age(y) (Mean ± SD)	BMI (kg/m^2^) ^1^ (Mean ± SD)	Baseline Vitamin D Level (nmol/L) (Mean ± SD)	Study Duration	Supplementation	Vitamin D Type	Dose & Frequency	Outcomes ^2^
Baziar 2014 [21]	Iran	41/40	33.3	6.9	T: 50.34 ± 6.71 C: 52.75 ± 6.34	T: 27.33 ± 1.64 C: 27.25 ± 1.35	T: 37.26 ± 15.21 C: 40.30 ± 14.43	8 weeks	Vit D	VD3	50,000 IU/week	①②③④
Maggi 2014 [28]	Italy	14/16	23.3	0.0	69	29	T: 27.79 ± 10.81 C: 33.73 ± 17.13	24 weeks	Vit D	VD3	300,000 IU once	①②
Anyanwu 2016 [20]	Nigeria	17/16	57.6	21.4	T: 52.5 ± 2.2 C: 51.1 ± 1.9	NR ^1^	T: 17.9 ± 2.3 C: 19.2 ± 5.5	12 weeks	Vit D	VD3	3000 IU/day	①②
Eftekhari 2011 [22]	Iran	35/35	50.0	0.0	T: 53.8 ± 8.9 C: 52.4 ± 7.8	T: 28.3 ±4.4 C: 27.0 ± 3.4	T: 112.6 ± 83.5 C: 100.1 ± 77.7	12 weeks	Vit D	VD3	20 IU/day	①②③④
Ghavamzadeh 2014 [23]	Iran	26/25	58.8	57.5	T: 52.26 ± 2.09 C: 49.28 ± 2.00	T: 28.9 ± 0.86 C: 27.9 ± 0.93	T: 21.46 ± 4.65 C: 22.16 ± 5.32	14 weeks	Vit D	VD3	400 IU/day	①③
Jorde 2009 [24]	Norway	16/16	43.8	11.1	T: 57.7 ± 9.7 C: 54.8 ± 5.9	T: 32.8 ± 6.8 C: 31.3 ± 6.3	T: 60.0 ± 14.0 C: 58.5 ± 21.0	6 months	Vit D	VD3	40,000 IU/week	①②③④
Kampmann 2014 [25]	Denmark	7/8	53.3	6.3	T: 61.6 ± 4.4 C: 57 ± 4.5	T: 35.3 ± 2.9 C: 32.4 ± 2.0	T: 31.0 ± 4.9 C: 34.8 ± 3.8	12 weeks	Vit D	VD3	11,200 IU/day × 2 weeks, then 5600 IU/day × 10 weeks	①②③⑤
Krul-Poel 2015 [27]	The Netherlands	129/132	34.9	5.1	T: 67 ± 8 C: 67 ± 9	T: 28.7 ± 4.6 C: 28.5 ± 4.5	T: 60.6 ± 23.3 C: 59.1 ± 23.2	6 months	Vit D	VD3	50,000 IU/month	①②③
Nasri 2014 [29]	Iran	30/30	71.7	0.0	55 ± 10.7	NR	T: 83.9 ± 52 C: 105.7 ± 64	12 weeks	Vit D	VD3	50,000 IU/week	①③
Ryu 2014 a [32]	Korea	32/30	NR	23.5	T: 54.5 ± 7.4 C: 56.7 ± 7.9	T: 24.4 ± 5.0 C: 25.3 ± 3.4	T: 32.0 ± 7.8 C: 27.8 ± 6.8	6 months	Vit D + Ca	VD3	2000 IU/day	①②③
Sadiya 2015 [34]	UAE^1^	43/39	81.6	5.7	T: 49 ± 8 C: 48 ± 8	T: 37.9 ± 6.1 C: 37.6± 7.7	T: 28.5 ± 9.2 C: 30.5 ± 11.3	6 months	Vit D	VD3	6000 IU/day × 3 months, then 3000 IU/day × 3 months	①②③
Yousefi 2014 [38]	Iran	28/30	37.9	10.8	T: 50.03 C: 49.90	T: 27.94 ± 0.92 C: 28.75 ± 0.95	T: 40.43 ± 4.97 C: 38.06 ± 5.77	2 months	Vit D	NR	4000 IU/day	①②③④⑤
Tabesh 2014 [36]	Iran	29/30	50.0	1.7	T: 50.2 ± 6.6 C: 51.0 ± 6.1	T: 30.5 ± 5.3 C: 30.3 ± 3.8	T: 28.0 ± 13.9 C: 45.7 ± 16.4	2 months	Vit D	VD3	50,000 U/week	④
Witham 2010 [37]	UK ^1^	19/21	32.8	2.4	T: 65.3 ± 11.1 C: 66.7 ± 9.7	T: 31.1 ± 6.7 C: 33.3 ± 7.1	T: 41 ± 14 C: 45 ± 17	4 months	Vit D	VD3	100,000 IU once	①③④
Ryu 2014 b [33]	Korea	64/65	50.0	18.4	T:54.8 ± 7.6 C:55.9 ± 8.1	T: 25.0 ± 3.3 C: 25.6 ± 3.6	T: 28.08 ± 13.26 C: 26.26 ± 10.14	6 months	Vit D +Ca	VD3	2000 IU/day	①②③
Punthakee 2012 [30]	33 countries	607/614	40.9	0.9	T: 66.7 ± 6.7 C: 66.6 ± 6.3	T: 30.6 ± 5.3 C: 30.7 ± 5.3	NR	4 months	Vit D	VD3	1000 IU/day	②③
Sugden 2008 [35]	UK ^1^	17/17	47.1	21.0	T: 64.9 ± 10.3 C: 63.5 ± 9.5	T: 31.7 ±6.4 C: 31.7 ± 6.5	T: 40.2 ± 10.3 C: 36.4 ± 8.5	2 months	Vit D	VD2	100,000 IU once	①③
Rashidi 2016 [31]	Iran	48/46	41.7	13.0	47	T: 28.08 ± 3.46 C: 28.65 ± 2.9	NR	3 months	Vit D	NR	50,000 IU/2 weeks	③
Kim 2014 [26]	Korea	11/13	100.0	13.3	T: 73.27 ± 2.06 C: 70.08 ± 1.37	T: 24.08 ± 0.73 C: 23.72 ± 0.68	T: 27.14 ± 4.68 C: 30.32 ± 7.28	3 months	Vit D	VD3	1200 IU/day	③④⑤
Zhou 2015 [39]	China	31/31	38.7	9.7	58.85 ± 6.18	T: 25.05 ± 3.30 C: 24.09 ± 3.77	T: 32.21 ± 21.76 C: 34.58 ± 20.18	3 months	Vit D	VD3	1000 IU/day	①③

^1^ Abbreviations: T: treatment group; C: control group; NR: not reported; UAE: The United Arab Emirates; UK: United Kingdom; BMI: body mass index.^2^ ① serum vitamin D levels; ② fasting blood glucose; ③ HbA1c; ④ HOMA-IR; ⑤ fasting serum insulin.

**Table 2:** Subgroup analyses.

Subgroups	Serum Vitamin D			FBG ^1^				HbA1c ^1^			HOMA-IR ^1^			Fasting Insulin		
	WMD ^1^	95%CI	*p*	WMD	95%CI	*p*		WMD	95%CI	*p*	SMD	95%CI	*p*	SMD	95%CI	*p*
Ethnicity																
Middle Easterners	40.03	(27.71, 52.34)	<0.001	−10.43	(−14.80, −6.06)	<0.001		−0.36	(−0.87, 0.15)	0.170	−0.65	(−1.37, 0.08)	0.081	−1.48	(−3.59, 0.63)	0.170
Other Asians	32.23	(22.72, 41.73)	<0.001	−1.83	(−7.80, 4.15)	0.549		−0.18	(−0.77, 0.42)	0.565	−1.59	(−2.42, −0.76)	<0.001	−1.80	(−2.66, −0.95)	<0.001
Other ethnicities	30.72	(12.25, 49.19)	0.001	1.67	(−1.50, 4.85)	0.301		0.17	(0.00, 0.33)	0.044	0.01	(−0.45, 0.47)	0.961	1.67	(0.47, 2.87)	0.006
BMI ^1^																
Normal ^2^	38.20	(27.89, 48.51)	<0.001	−5.21	(−11.76, 1.34)	0.119		0.16	(−0.13, 0.45)	0.274	−1.59	(−2.42, −0.76)	<0.001	−1.80	(−2.66, −0.95)	<0.001
Overweight ^2^	33.01	(23.94, 42.09)	<0.001	−5.71	(−14.09, 2.66)	0.181		−0.32	(−0.70, 0.06)	0.102	−0.84	(−1.78, 0.11)	0.084	−1.48	(−3.59, 0.63)	0.170
Obese ^2^	39.34	(18.71, 59.97)	<0.001	0.64	(−3.32, 4.60)	0.751		0.22	(0.05, 0.38)	0.010	−0.04	(−0.38, 0.30)	0.825	1.67	(0.47, 2.87)	0.006
Dose																
≤2000 IU/day	25.16	(18.16, 32.16)	<0.001	0.35	(−3.18, 3.89)	0.844		−0.21	(−0.53, 0.11)	0.189	−0.50	(−1.35, 0.35)	0.249	−1.80	(−2.66, −0.95)	<0.001
>2000 IU/day	48.45	(29.94, 66.97)	<0.001	−8.70	(−12.96, −4.44)	<0.001		0.05	(−0.41, 0.51)	0.832	−0.64	(−1.42, 0.14)	0.107	−0.50	(−2.44, 1.45)	0.617
Duration																
≤3 m	35.51	(18.84, 52.18)	<0.001	−8.44	(−12.72, −4.15)	<0.001		−0.11	(−0.42, 0.21)	0.590	−0.81	(−1.49, −0.13)	0.019	----	----	----
>3 m	32.61	(24.951, 40.271)	<0.001	2.04	(-0.94, 5.02)	0.180		−0.12	(−0.54, 0.31)	0.509	0.01	(−0.45, 0.47)	0.961	----	----	----
Baseline 25(OH)D																
<50 nmol/L	31.65	(21.31, 41.99)	<0.001	−5.77	(−10.48, −1.05)	0.017		−0.11	(−0.47, 0.26)	0.563	−0.81	(−1.51, −0.11)	0.024	----	----	----
50–75 nmol/L	48.33	(29.46, 67.21)	<0.001	3.37	(−1.81, 8.54)	0.202		−0.00	(−0.14, 0.14)	1.000	0.03	(−0.67, 0.72)	0.941	----	----	----
>75 nmol/L	32.98	(−39.64, 105.06)	0.373	−18.00	(−43.24, 7.24)	0.162		−0.39	(−0.78, 0.00)	0.052	−0.08	(−0.55, 0.39)	0.737	----	----	----
Baseline HbA1c ^1^																
≤7%	45.27	(25.39, 65.15)	<0.001	−4.09	(−15.44, 7.27)	0.481		−0.17	(−0.86, 0.52)	0.635	−0.27	(−0.65, 0.11)	0.160	0.55	(−1.49,2.59)	0.53
>7%	29.86	(18.22, 41.49)	<0.001	−3.53	(−9.42, 2.35)	0.240		−0.08	(−0.34, 0.18)	0.548	−0.65	(−1.83, 0.54)	0.286	−2.57	(−3.27, −1.87)	<0.001
Overall	33.98	(24.60, 43.37)	<0.001	−3.59	(−7.94, 0.76)	0.105		−0.11	(−0.35, 0.13)	0.381	−0.58	(−1.11, −0.05)	0.033	−0.83	(−2.31, 0.64)	0.268

^1^ Abbreviations: WMD: weighted mean difference; SMD: standardized mean difference; FBG: fasting blood glucose; HbA1c: hemoglobin A1c; HOMA-IR: homeostatic model assessment of insulin resistance; BMI: Body mass index. ^2^ normal: 18.5–24.9kg/m^2^; overweight: 25.0–29.9 kg/m^2^; obese: ≥30.0 kg/m^2^. “----”: subgroup inapplicable to this outcome.

**Table 3:** Evidence quality rated using the GRADE approach.

Outcomes	No. of Studies	Limitations	Inconsistency	Indirectness	Imprecision	Publication Bias	Evidence Quality	
Serum vitamin D levels	16	Serious ^2,3,4^	Serious ^5^	Not serious	Not serious	Not found	⊕⊕⊝⊝	Low
FBG ^1^	13	Serious ^2,3,4^	Serious ^5^	Not serious	Serious ^6^	Not found	⊕⊝⊝⊝	Very low
HbA1c ^1^	15	Serious ^3,4^	Serious ^5^	Not serious	Serious ^6^	Not found	⊕⊝⊝⊝	Very low
HOMA-IR ^1^	7	Serious ^3,4^	Serious ^5^	Not serious	Not serious	Not assessed ^7^	⊕⊕⊝⊝	Low
Fasting serum insulin	4	Serious ^4^	Serious ^5^	Not serious	Serious ^6^	Not assessed ^7^	⊕⊝⊝⊝	Very low

^1^ Abbreviations: FBG: fasting blood glucose; HbA1c: hemoglobin A1c; HOMA-IR: homeostasis model assessment of insulin resistance. ^2^ Had single-blind randomized controlled trial design; ^3^ had reporting bias; ^4^ had attrition bias. ^5^ A significant heterogeneity was observed in this meta-analysis. ^6^ Wide confidence interval, including values in favour of the experimental group and values in favour of the control group. ^7^ Not assessed because a limited number of studies were included in the meta-analyses on HOMA-IR and fasting serum insulin.
